# 2,4-Bis(4-chloro­phen­yl)-3-aza­bicyclo­[3.3.1]nonan-9-one

**DOI:** 10.1107/S160053680900590X

**Published:** 2009-02-25

**Authors:** P. Parthiban, V. Ramkumar, Min Sung Kim, S. Kabilan, Yeon Tae Jeong

**Affiliations:** a Division of Image Science and Information Engineering, Pukyong National University, Busan 608 739, Republic of Korea; b Department of Chemistry, IIT Madras, Chennai, TamilNadu, India; c Department of Chemistry, Annamalai University, Annamalai Nagar, TamilNadu, India

## Abstract

In the mol­ecular structure of the title compound, C_20_H_19_Cl_2_NO, the mol­ecule exists in a twin-chair conformation with equatorial dispositions of the 4-chloro­phenyl groups on both sides of the secondary amino group; the dihedral angle between the aromatic ring planes is 31.33 (3)°. The crystal structure is stabilized by N—H⋯O inter­actions, leading to chains of molecules.

## Related literature

For the biological activity of diterpenoid/norditerpenoid alkaloids, see: Hardick *et al.* (1996 ▶); Jeyaraman *et al.* (1981 ▶). For similiar structures, see: Parthiban *et al.* (2008*a* ▶,*b* ▶,*c* ▶,*d* ▶,*e* ▶). For puckering parameters, see: Cremer & Pople (1975 ▶).
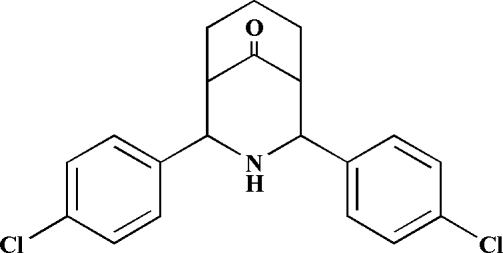


## Experimental

### 

#### Crystal data

C_20_H_19_Cl_2_NO
*M* *_r_* = 360.26
Monoclinic, 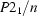

*a* = 16.2589 (4) Å
*b* = 6.8983 (2) Å
*c* = 18.1291 (5) Å
β = 116.149 (1)°
*V* = 1825.23 (9) Å^3^
*Z* = 4
Mo *K*α radiation
μ = 0.36 mm^−1^
*T* = 298 K
0.42 × 0.38 × 0.25 mm

#### Data collection

Bruker APEXII CCD area-detector diffractometer
Absorption correction: multi-scan (*SADABS*; Bruker, 1999 ▶) *T*_min_ = 0.863, *T*_max_ = 0.915
15050 measured reflections
4997 independent reflections
3241 reflections with *I* > 2σ(*I*)
*R*_int_ = 0.021

#### Refinement

*R*[*F* ^2^ > 2σ(*F* ^2^)] = 0.046
*wR*(*F* ^2^) = 0.135
*S* = 1.02
4997 reflections
221 parameters
H atoms treated by a mixture of independent and constrained refinement
Δρ_max_ = 0.38 e Å^−3^
Δρ_min_ = −0.42 e Å^−3^

### 

Data collection: *APEX2* (Bruker, 2004 ▶); cell refinement: *SAINT-Plus* (Bruker, 2004 ▶); data reduction: *SAINT-Plus*; program(s) used to solve structure: *SHELXS97* (Sheldrick, 2008 ▶); program(s) used to refine structure: *SHELXL97* (Sheldrick, 2008 ▶); molecular graphics: *ORTEP-3* (Farrugia, 1997 ▶); software used to prepare material for publication: *SHELXL97*.

## Supplementary Material

Crystal structure: contains datablocks global, I. DOI: 10.1107/S160053680900590X/bq2124sup1.cif

Structure factors: contains datablocks I. DOI: 10.1107/S160053680900590X/bq2124Isup2.hkl

Additional supplementary materials:  crystallographic information; 3D view; checkCIF report

## Figures and Tables

**Table 1:** Hydrogen-bond geometry (Å, °)

*D*—H⋯*A*	*D*—H	H⋯*A*	*D*⋯*A*	*D*—H⋯*A*
N1—H1*A*⋯O1^i^	0.85 (2)	2.31 (2)	3.1202 (18)	160.2 (18)

Symmetry code: (i) 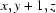.

**Fig. 1.**
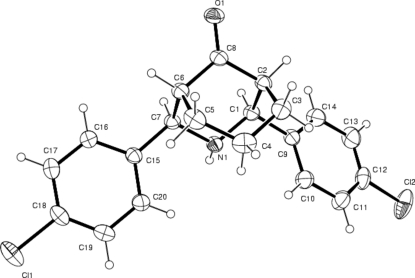
ORTEP of the molecule with atoms represented as 30% probability ellipsoids.

**Fig. 2.**
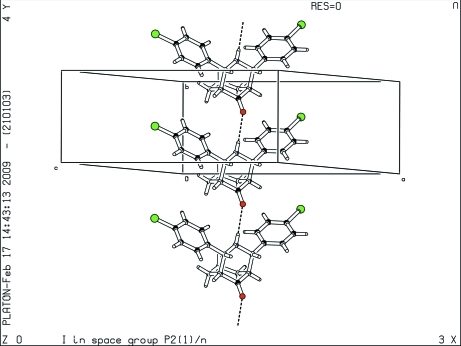
Packing of molecules via N-H..O bond.

## supplementary crystallographic information

### Comment

The widespread diterpenoid/norditerpenoid alkaloids posses the 3-azabicyclo [3.3.1]nonane pharmacophore, and as a consequence, the above alkaloids having broad spectrum of biological activities (Jeyaraman *et al.*, 1981; Hardick *et al.*, 1996). Hence, the synthesis and stereochemistry of 3-azabicyclononan-9-ones are more important in recent days (Parthiban *et al.*, 2008a,b,c,d,e). A study of torsion angles, asymmetry parameters and least-squares plane calculation shows that the piperidine ring adopts near ideal chair conformation with the deviation of ring atoms N1 and C8 from the C1/C2/C6/C7 plane by -0.642 (3)Å and 0.712 (3)Å, respectively, Q_T_=0.607 (2)Å, q(2)=0.044 (2)Å and q(3)=-0.606 (2)Å, θ=175.8 (2)°. whereas the cyclohexane ring deviate from the ideal chair conformation; the cyclohexane atoms C4 and C8 deviate from the C2/C3/C5/C6 plane by -0.557 (2)Å and 0.710 (3)Å, respectively, Q_T_=0.560 (2) Å, q(2)=0.117 (2)Å and, q(3)=-0.548 (2)Å, θ=167.9 (2)°. (Cremer & Pople, 1975). Hence, the title compound C_20_H_19_Cl_2_NO, exists in double chair conformation with equatorial dispositions of the *para* chlorophenyl groups with the torsion angles of C8—C2—C1—C9 and C8—C6—C7—C15 are 177.88 (4)° and -179.01 (4)°, respectively. The aryl groups are oriented at an angle of 31.33 (3)° to each other.

### Experimental

In a warm solution of ammonium acetate (0.075 mol) in 50 ml of absolute ethanol, a mixture of cyclohexanone (0.05 mol) and *para* chlorobenzaldehyde (0.1 mol) was added and gently warmed with stirring on a hot plate till the yellow color was formed during the mixing of the reactants and cooled to room temperature. Then 50 ml of ether was added and allowed to stir over night at room temperature. Thus the obtained crude azabicyclic ketone was separated by filtration and washed with 1:5 ethanol-ether mixture till the solid became colorless. Recrystallization of the compound from ethanol gave X-ray diffraction quality crystals of 2,4-bis(4-chlorophenyl)-3-azabicyclo[3.3.1]nonan-9-one.

### Refinement

Nitrogen H atoms were located in a difference Fourier map and refined isotropically. Other hydrogen atoms were fixed geometrically and allowed to ride on the parent carbon atoms, with aromatic C—H =0.93Å, aliphatic C—H =0.98Å and methylene C—H=0.97Å. The displacement parameters were set for phenyl, methylene and aliphatic H atoms at *U*_iso_(H)=1.2*U*_eq_(C).

### Crystal data

**Table:**

C_20_H_19_Cl_2_NO	*F*(000) = 752
*M**_r_* = 360.26	*D*_x_ = 1.311 Mg m^−^^3^
Monoclinic, *P*2_1_/*n*	Mo *K*α radiation, λ = 0.71073 Å
Hall symbol: -P 2yn	Cell parameters from 4308 reflections
*a* = 16.2589 (4) Å	θ = 2.8–26.3°
*b* = 6.8983 (2) Å	µ = 0.36 mm^−^^1^
*c* = 18.1291 (5) Å	*T* = 298 K
β = 116.149 (1)°	Rectangular, colourless
*V* = 1825.23 (9) Å^3^	0.42 × 0.38 × 0.25 mm
*Z* = 4	

### Data collection

**Table:**

Bruker APEXII CCD area-detector diffractometer	4997 independent reflections
Radiation source: fine-focus sealed tube	3241 reflections with *I* > 2σ(*I*)
graphite	*R*_int_ = 0.021
φ and ω scans	θ_max_ = 29.8°, θ_min_ = 2.3°
Absorption correction: multi-scan (*SADABS*; Bruker, 1999)	*h* = −21→20
*T*_min_ = 0.863, *T*_max_ = 0.915	*k* = −9→9
15050 measured reflections	*l* = −15→25

### Refinement

**Table:**

Refinement on *F*^2^	Primary atom site location: structure-invariant direct methods
Least-squares matrix: full	Secondary atom site location: difference Fourier map
*R*[*F*^2^ > 2σ(*F*^2^)] = 0.046	Hydrogen site location: inferred from neighbouring sites
*wR*(*F*^2^) = 0.135	H atoms treated by a mixture of independent and constrained refinement
*S* = 1.02	*w* = 1/[σ^2^(*F*_o_^2^) + (0.0602*P*)^2^ + 0.4485*P*] where *P* = (*F*_o_^2^ + 2*F*_c_^2^)/3
4997 reflections	(Δ/σ)_max_ < 0.001
221 parameters	Δρ_max_ = 0.38 e Å^−^^3^
0 restraints	Δρ_min_ = −0.42 e Å^−^^3^

### Special details

**Table:**

Geometry. All e.s.d.'s (except the e.s.d. in the dihedral angle between two l.s. planes)are estimated using the full covariance matrix. The cell e.s.d.'s are takeninto account individually in the estimation of e.s.d.'s in distances, anglesand torsion angles; correlations between e.s.d.'s in cell parameters are onlyused when they are defined by crystal symmetry. An approximate (isotropic)treatment of cell e.s.d.'s is used for estimating e.s.d.'s involving l.s. planes.
Refinement. Refinement of *F*^2^ against ALL reflections. The weighted *R*-factor *wR* andgoodness of fit *S* are based on *F*^2^, conventional *R*-factors *R* are basedon *F*, with *F* set to zero for negative *F*^2^. The threshold expression of*F*^2^ > σ(*F*^2^) is used only for calculating *R*-factors(gt) *etc*. and is not relevant to the choice of reflections for refinement. *R*-factors based on *F*^2^ are statistically about twice as large as those based on *F*, and *R*- factors based on ALL data will be even larger.

### Fractional atomic coordinates and isotropic or equivalent isotropic displacement parameters (Å^2^)

**Table:**

	*x*	*y*	*z*	*U*_iso_*/*U*_eq_	
C1	0.33305 (11)	0.1260 (2)	0.01759 (10)	0.0384 (3)	
H1	0.3907	0.1019	0.0665	0.046*	
C2	0.29289 (11)	−0.0738 (2)	−0.02171 (10)	0.0410 (4)	
H2	0.3380	−0.1414	−0.0345	0.049*	
C3	0.20110 (12)	−0.0678 (3)	−0.09929 (11)	0.0533 (4)	
H3A	0.2079	0.0135	−0.1399	0.064*	
H3B	0.1864	−0.1977	−0.1219	0.064*	
C4	0.12129 (12)	0.0082 (3)	−0.08496 (12)	0.0581 (5)	
H4A	0.1268	0.1478	−0.0779	0.070*	
H4B	0.0643	−0.0190	−0.1330	0.070*	
C5	0.11811 (12)	−0.0827 (3)	−0.00979 (12)	0.0534 (5)	
H5A	0.0944	−0.2136	−0.0235	0.064*	
H5B	0.0756	−0.0096	0.0037	0.064*	
C6	0.21147 (11)	−0.0905 (2)	0.06658 (11)	0.0418 (4)	
H6	0.2052	−0.1688	0.1090	0.050*	
C7	0.25223 (11)	0.1109 (2)	0.10384 (10)	0.0375 (3)	
H7	0.3106	0.0893	0.1524	0.045*	
C8	0.27891 (11)	−0.1870 (2)	0.04285 (10)	0.0390 (4)	
C9	0.35398 (11)	0.2533 (2)	−0.03978 (10)	0.0414 (4)	
C10	0.29057 (13)	0.3777 (3)	−0.09592 (12)	0.0544 (5)	
H10	0.2316	0.3836	−0.1000	0.065*	
C11	0.31400 (15)	0.4943 (3)	−0.14645 (12)	0.0615 (5)	
H11	0.2710	0.5776	−0.1841	0.074*	
C12	0.40091 (16)	0.4852 (3)	−0.14022 (12)	0.0584 (5)	
C13	0.46439 (15)	0.3618 (3)	−0.08633 (14)	0.0654 (6)	
H13	0.5228	0.3545	−0.0835	0.078*	
C14	0.44093 (13)	0.2474 (3)	−0.03588 (13)	0.0555 (5)	
H14	0.4845	0.1646	0.0015	0.067*	
C15	0.18866 (11)	0.2164 (2)	0.13081 (10)	0.0385 (3)	
C16	0.17785 (12)	0.1464 (3)	0.19803 (11)	0.0473 (4)	
H16	0.2125	0.0407	0.2271	0.057*	
C17	0.11692 (13)	0.2301 (3)	0.22247 (11)	0.0528 (4)	
H17	0.1097	0.1804	0.2670	0.063*	
C18	0.06701 (12)	0.3877 (3)	0.18023 (12)	0.0509 (4)	
C19	0.07748 (13)	0.4646 (3)	0.11480 (12)	0.0540 (5)	
H19	0.0446	0.5740	0.0877	0.065*	
C20	0.13769 (12)	0.3765 (2)	0.08997 (11)	0.0471 (4)	
H20	0.1440	0.4259	0.0450	0.057*	
Cl1	−0.01108 (4)	0.49239 (10)	0.21027 (4)	0.0838 (2)	
Cl2	0.43337 (5)	0.64178 (11)	−0.19813 (4)	0.0956 (3)	
N1	0.27045 (9)	0.2229 (2)	0.04419 (9)	0.0401 (3)	
O1	0.31840 (9)	−0.33704 (16)	0.07301 (8)	0.0515 (3)	
H1A	0.2917 (13)	0.334 (3)	0.0645 (12)	0.058 (6)*	

### Atomic displacement parameters (Å^2^)

**Table:**

	*U*^11^	*U*^22^	*U*^33^	*U*^12^	*U*^13^	*U*^23^
C1	0.0379 (8)	0.0331 (8)	0.0479 (9)	−0.0024 (6)	0.0223 (7)	0.0015 (7)
C2	0.0440 (9)	0.0329 (8)	0.0532 (9)	−0.0007 (7)	0.0278 (8)	−0.0020 (7)
C3	0.0591 (11)	0.0489 (10)	0.0498 (10)	−0.0103 (9)	0.0221 (9)	−0.0051 (8)
C4	0.0413 (10)	0.0552 (12)	0.0634 (12)	−0.0046 (8)	0.0100 (9)	−0.0021 (10)
C5	0.0407 (9)	0.0473 (10)	0.0750 (13)	−0.0100 (8)	0.0279 (9)	−0.0112 (9)
C6	0.0462 (9)	0.0304 (8)	0.0578 (10)	−0.0030 (7)	0.0312 (8)	0.0018 (7)
C7	0.0387 (8)	0.0317 (7)	0.0458 (9)	0.0003 (6)	0.0218 (7)	0.0016 (7)
C8	0.0401 (8)	0.0273 (7)	0.0514 (9)	−0.0059 (6)	0.0219 (7)	−0.0053 (7)
C9	0.0470 (9)	0.0327 (8)	0.0530 (9)	−0.0049 (7)	0.0298 (8)	−0.0023 (7)
C10	0.0526 (11)	0.0557 (11)	0.0635 (11)	0.0042 (9)	0.0334 (9)	0.0110 (9)
C11	0.0770 (14)	0.0560 (12)	0.0556 (11)	0.0002 (10)	0.0331 (11)	0.0112 (9)
C12	0.0821 (14)	0.0544 (11)	0.0527 (10)	−0.0244 (10)	0.0424 (10)	−0.0081 (9)
C13	0.0637 (13)	0.0668 (13)	0.0862 (15)	−0.0121 (11)	0.0517 (12)	0.0008 (12)
C14	0.0514 (11)	0.0483 (10)	0.0781 (13)	−0.0017 (8)	0.0387 (10)	0.0053 (9)
C15	0.0397 (8)	0.0334 (8)	0.0445 (8)	−0.0023 (6)	0.0205 (7)	−0.0035 (7)
C16	0.0531 (10)	0.0452 (10)	0.0471 (9)	0.0052 (8)	0.0254 (8)	0.0035 (8)
C17	0.0584 (11)	0.0601 (12)	0.0475 (10)	−0.0022 (9)	0.0305 (9)	−0.0062 (9)
C18	0.0442 (10)	0.0534 (11)	0.0576 (11)	−0.0012 (8)	0.0249 (9)	−0.0186 (9)
C19	0.0515 (11)	0.0435 (10)	0.0654 (12)	0.0096 (8)	0.0243 (9)	−0.0027 (9)
C20	0.0534 (10)	0.0394 (9)	0.0528 (10)	0.0025 (8)	0.0274 (8)	0.0022 (8)
Cl1	0.0721 (4)	0.0962 (5)	0.0986 (5)	0.0135 (3)	0.0519 (3)	−0.0262 (4)
Cl2	0.1246 (6)	0.1079 (5)	0.0667 (4)	−0.0502 (4)	0.0535 (4)	0.0061 (3)
N1	0.0490 (8)	0.0274 (6)	0.0524 (8)	−0.0046 (6)	0.0302 (7)	−0.0020 (6)
O1	0.0613 (8)	0.0313 (6)	0.0686 (8)	0.0042 (5)	0.0347 (7)	0.0037 (6)

### Geometric parameters (Å, °)

**Table:**

C1—N1	1.466 (2)	C9—C10	1.382 (3)
C1—C9	1.511 (2)	C9—C14	1.385 (2)
C1—C2	1.557 (2)	C10—C11	1.393 (3)
C1—H1	0.9800	C10—H10	0.9300
C2—C8	1.505 (2)	C11—C12	1.368 (3)
C2—C3	1.535 (2)	C11—H11	0.9300
C2—H2	0.9800	C12—C13	1.363 (3)
C3—C4	1.524 (3)	C12—Cl2	1.7422 (18)
C3—H3A	0.9700	C13—C14	1.383 (3)
C3—H3B	0.9700	C13—H13	0.9300
C4—C5	1.522 (3)	C14—H14	0.9300
C4—H4A	0.9700	C15—C20	1.383 (2)
C4—H4B	0.9700	C15—C16	1.392 (2)
C5—C6	1.540 (3)	C16—C17	1.378 (2)
C5—H5A	0.9700	C16—H16	0.9300
C5—H5B	0.9700	C17—C18	1.371 (3)
C6—C8	1.499 (2)	C17—H17	0.9300
C6—C7	1.559 (2)	C18—C19	1.377 (3)
C6—H6	0.9800	C18—Cl1	1.7433 (17)
C7—N1	1.462 (2)	C19—C20	1.385 (2)
C7—C15	1.511 (2)	C19—H19	0.9300
C7—H7	0.9800	C20—H20	0.9300
C8—O1	1.2137 (19)	N1—H1A	0.85 (2)
			
N1—C1—C9	111.16 (13)	O1—C8—C6	123.91 (15)
N1—C1—C2	110.05 (12)	O1—C8—C2	124.16 (15)
C9—C1—C2	112.08 (13)	C6—C8—C2	111.92 (13)
N1—C1—H1	107.8	C10—C9—C14	118.01 (16)
C9—C1—H1	107.8	C10—C9—C1	123.14 (15)
C2—C1—H1	107.8	C14—C9—C1	118.85 (16)
C8—C2—C3	107.89 (13)	C9—C10—C11	120.76 (18)
C8—C2—C1	106.25 (13)	C9—C10—H10	119.6
C3—C2—C1	115.97 (14)	C11—C10—H10	119.6
C8—C2—H2	108.8	C12—C11—C10	119.42 (19)
C3—C2—H2	108.8	C12—C11—H11	120.3
C1—C2—H2	108.8	C10—C11—H11	120.3
C4—C3—C2	114.21 (15)	C13—C12—C11	121.07 (17)
C4—C3—H3A	108.7	C13—C12—Cl2	118.99 (16)
C2—C3—H3A	108.7	C11—C12—Cl2	119.86 (18)
C4—C3—H3B	108.7	C12—C13—C14	119.24 (19)
C2—C3—H3B	108.7	C12—C13—H13	120.4
H3A—C3—H3B	107.6	C14—C13—H13	120.4
C5—C4—C3	112.21 (16)	C13—C14—C9	121.49 (19)
C5—C4—H4A	109.2	C13—C14—H14	119.3
C3—C4—H4A	109.2	C9—C14—H14	119.3
C5—C4—H4B	109.2	C20—C15—C16	117.94 (15)
C3—C4—H4B	109.2	C20—C15—C7	123.06 (14)
H4A—C4—H4B	107.9	C16—C15—C7	118.96 (14)
C4—C5—C6	114.24 (14)	C17—C16—C15	121.48 (17)
C4—C5—H5A	108.7	C17—C16—H16	119.3
C6—C5—H5A	108.7	C15—C16—H16	119.3
C4—C5—H5B	108.7	C18—C17—C16	119.03 (17)
C6—C5—H5B	108.7	C18—C17—H17	120.5
H5A—C5—H5B	107.6	C16—C17—H17	120.5
C8—C6—C5	108.27 (14)	C17—C18—C19	121.29 (16)
C8—C6—C7	107.15 (12)	C17—C18—Cl1	119.16 (15)
C5—C6—C7	114.88 (14)	C19—C18—Cl1	119.55 (15)
C8—C6—H6	108.8	C18—C19—C20	118.97 (17)
C5—C6—H6	108.8	C18—C19—H19	120.5
C7—C6—H6	108.8	C20—C19—H19	120.5
N1—C7—C15	112.03 (12)	C15—C20—C19	121.25 (17)
N1—C7—C6	109.68 (13)	C15—C20—H20	119.4
C15—C7—C6	110.42 (12)	C19—C20—H20	119.4
N1—C7—H7	108.2	C7—N1—C1	113.48 (12)
C15—C7—H7	108.2	C7—N1—H1A	109.2 (14)
C6—C7—H7	108.2	C1—N1—H1A	110.1 (13)
			
N1—C1—C2—C8	−57.87 (16)	C1—C9—C10—C11	178.93 (17)
C9—C1—C2—C8	177.89 (13)	C9—C10—C11—C12	0.0 (3)
N1—C1—C2—C3	61.99 (18)	C10—C11—C12—C13	1.1 (3)
C9—C1—C2—C3	−62.25 (18)	C10—C11—C12—Cl2	−175.72 (16)
C8—C2—C3—C4	53.9 (2)	C11—C12—C13—C14	−1.5 (3)
C1—C2—C3—C4	−65.1 (2)	Cl2—C12—C13—C14	175.27 (16)
C2—C3—C4—C5	−46.3 (2)	C12—C13—C14—C9	1.0 (3)
C3—C4—C5—C6	45.7 (2)	C10—C9—C14—C13	0.0 (3)
C4—C5—C6—C8	−53.0 (2)	C1—C9—C14—C13	−179.45 (18)
C4—C5—C6—C7	66.6 (2)	N1—C7—C15—C20	14.1 (2)
C8—C6—C7—N1	57.05 (17)	C6—C7—C15—C20	−108.49 (17)
C5—C6—C7—N1	−63.26 (17)	N1—C7—C15—C16	−168.34 (15)
C8—C6—C7—C15	−179.02 (13)	C6—C7—C15—C16	69.09 (19)
C5—C6—C7—C15	60.67 (18)	C20—C15—C16—C17	1.5 (3)
C5—C6—C8—O1	−118.50 (18)	C7—C15—C16—C17	−176.24 (16)
C7—C6—C8—O1	117.07 (17)	C15—C16—C17—C18	−1.0 (3)
C5—C6—C8—C2	62.45 (17)	C16—C17—C18—C19	−0.8 (3)
C7—C6—C8—C2	−61.98 (17)	C16—C17—C18—Cl1	179.30 (14)
C3—C2—C8—O1	118.08 (18)	C17—C18—C19—C20	2.0 (3)
C1—C2—C8—O1	−116.94 (17)	Cl1—C18—C19—C20	−178.05 (14)
C3—C2—C8—C6	−62.87 (17)	C16—C15—C20—C19	−0.2 (3)
C1—C2—C8—C6	62.11 (17)	C7—C15—C20—C19	177.44 (16)
N1—C1—C9—C10	−35.0 (2)	C18—C19—C20—C15	−1.5 (3)
C2—C1—C9—C10	88.57 (19)	C15—C7—N1—C1	179.14 (13)
N1—C1—C9—C14	144.36 (16)	C6—C7—N1—C1	−57.87 (17)
C2—C1—C9—C14	−92.02 (19)	C9—C1—N1—C7	−176.47 (13)
C14—C9—C10—C11	−0.5 (3)	C2—C1—N1—C7	58.76 (17)

### Hydrogen-bond geometry (Å, °)

**Table:**

*D*—H···*A*	*D*—H	H···*A*	*D*···*A*	*D*—H···*A*
N1—H1A···O1^i^	0.85 (2)	2.31 (2)	3.1202 (18)	160.2 (18)

Symmetry codes: (i) *x*, *y*+1, *z*.
